# Reply to: Critical COVID-19 and neurological dysfunction - a direct comparative analysis between SARS-CoV-2 and other infectious pathogens

**DOI:** 10.62675/2965-2774.20240180-en

**Published:** 2024-10-21

**Authors:** Ana Teixeira-Vaz, José Artur Paiva

**Affiliations:** 1 Unidade Local de Saúde de São João Physical Medicine and Rehabilitation Department Porto Portugal Physical Medicine and Rehabilitation Department, Unidade Local de Saúde de São João - Porto, Portugal.; 1 Unidade Local de Saúde de São João Intensive Care Department Porto Portugal Intensive Care Department, Unidade Local de Saúde de São João - Porto, Portugal.

To the Editor

The authors thank Dr. Villa^(1)^ for his interest in and praise for our study entitled "Critical COVID-19 and neurological dysfunction - a direct comparative analysis between SARS-CoV-2 and other infectious pathogens".^(2)^

The authors highlight that this study was a prospective comparative cohort study that included, in accordance with the sample size calculation, 27 patients with acute respiratory distress syndrome (ARDS) caused by severe acute respiratory syndrome coronavirus 2 (SARS-CoV-2) and 27 patients with ARDS caused by other pathogens (54 patients in total). The aim of this investigation was to assess whether SARS-CoV-2 was more frequently associated with signs of corticospinal tract dysfunction and other neurological signs, symptoms and syndromes during hospital stay than other pathogens causing severe respiratory failure. In our sample, 61% of patients with ARDS (irrespective of the causative pathogen) presented neurological complications, which is consistent with the findings of Huang et al.^(3)^ Patients with SARS-CoV-2 ARDS had a 1.98-fold greater relative risk (95%CI 1.23 - 3.26) of neurological dysfunction, which was present in 85% of SARS-CoV-2 patients (not almost all). The incidence of neurological dysfunction in coronavirus disease 2019 (COVID-19) patients has been widely reported in the literature, ranging from 10 to 90% in accordance with disease severity, required support and other clinical characteristics.^(4,5)^ In contrast, comparative studies regarding the prevalence and characteristics of neurological complications between patients with critical respiratory pathology caused by COVID-19 and those caused by other pathogens are scarse.

Although our group performed a 1-year follow-up analysis of critically ill COVID-19 survivors to evaluate its impact in the three domains of postintensive care syndrome as well as in functionality,^(6)^ this study, as reported in the Methods section of our paper, was exclusively performed during the patient's hospital stay (clinical evaluations were performed in the first 24 - 72 hours after ventilatory weaning, with reevaluations performed every 24 - 72 hours, until three observations were completed), with the inherent particularities of an ICU setting investigation.

The authors agree with Dr. Villa^(1)^ that further prospective studies to analyze the clinical characteristics and time course of neurological dysfunction after severe SARS-CoV-2 infection are warranted. Several systematic reviews with meta-analyses are available regarding this topic;^(7-9)^ however, randomized controlled trials are still necessary to define the best strategies to reduce disease burden.^(7)^

The authors also believe that closely examining neurological damage in critically ill patients (inclusively exceeding the SARS-CoV-2 population) is of fundamental importance. Indeed, all patients included in the study underwent daily neurological examinations during their stay in the ICU, in accordance with the standard of clinical practice of the Intensive Care Medicine Department of our center, supported by international guidelines.^(10)^

Regarding the points noted by Drs. Finsterer and Scorza^(11)^ and Magoon and Suresh,^(12)^ those topics have been addressed in their respective Letter replies.^(13,14)^

We acknowledge the limitations of our study, as reported in the Discussion section of our paper, which is the reason that the main take-home message of our paper is the need for systematic screening for neurological complications in ARDS patients, as moreover in those with COVID-19-associated acute respiratory failure.^(2)^

The authors thank Dr. Villa^(1)^ for this discussion related to our paper in a particular topic we all agree: the importance of evaluating and treating COVID-19-related neurological dysfunction.
