# Supplementary material for: Reply to: Critical COVID-19 and neurological dysfunction - a direct comparative analysis between SARS-CoV-2 and other infectious pathogens
Source: Crit Care Sci. 2024 Oct 21;36:e20240180en. doi: 10.62675/2965-2774.20240180-en (PMC11554298; doi:10.62675/2965-2774.20240180-en)
Supplement: Translation

# Translation: Resposta para: COVID-19 crítico e disfunção neurológica - uma análise comparativa direta entre o SARS-CoV-2 e outros patógenos infecciosos

**DOI:** 10.62675/2965-2774.20240180-pt

**Authors:** Ana Teixeira-Vaz, José Artur Paiva

**Affiliations:** 1 Porto Portugal Departamento de Medicina Física e Reabilitação, Unidade Local de Saúde de São João - Porto, Portugal.; 2 Porto Portugal Departamento de Terapia Intensiva, Unidade Local de Saúde de São João - Porto, Portugal.

Ao Editor

Os autores agradecem ao Dr. Villa^(1)^ por seu interesse e pelos elogios ao nosso estudo intitulado *Critical COVID-19 and neurological dysfunction - a direct comparative analysis between SARS-CoV-2 and other infectious pathogens*.^(2)^

Destacamos que se trata de um estudo de coorte prospectivo comparativo que incluiu, de acordo com o cálculo do tamanho da amostra, 27 pacientes com síndrome do desconforto respiratório agudo (SDRA) causada pelo coronavírus da síndrome respiratória aguda grave 2 (SARS-CoV-2) e 27 pacientes com SDRA causada por outros patógenos (54 pacientes no total). O objetivo desta pesquisa foi avaliar se o SARS-CoV-2 foi mais frequentemente associado a sinais de disfunção do trato corticoespinhal e outros sinais, sintomas e síndromes neurológicos durante a internação hospitalar do que outros patógenos que causam insuficiência respiratória grave. Em nossa amostra, 61% dos pacientes com SDRA (independentemente do patógeno causador) apresentaram complicações neurológicas, o que é consistente com os achados de Huang et al.^(3)^ Os pacientes com SDRA por SARS-CoV-2 tiveram um risco relativo 1,98 vez maior (IC95% 1,23 - 3,26) de disfunção neurológica, que estava presente em 85% dos pacientes com SARS-CoV-2 (não todos). A incidência de disfunção neurológica em pacientes com doença pelo coronavírus 2019 (COVID-19) foi amplamente relatada na literatura, variando de 10 a 90%, de acordo com a gravidade da doença, o suporte necessário e outras características clínicas.^(4,5)^ Por outro lado, são muito raros estudos comparativos sobre a prevalência e as características das complicações neurológicas entre pacientes com patologia respiratória crítica causada pela COVID-19 e aqueles causados por outros patógenos.

Embora nosso grupo tenha realizado uma análise de seguimento de 1 ano de sobreviventes críticos da COVID-19 para avaliar seu impacto nos três domínios da síndrome de cuidados pós-intensivos, bem como a funcionalidade,^(6)^ este estudo, conforme relatado na seção Métodos do nosso artigo, foi realizado exclusivamente durante a internação do paciente (as avaliações clínicas foram realizadas nas primeiras 24 a 72 horas após o desmame ventilatório, com reavaliações realizadas a cada 24 a 72 horas, até que fossem concluídas três observações), com as particularidades inerentes a uma investigação em ambiente de UTI.

Os autores concordam com o Dr. Villa^(1)^ que são necessários mais estudos prospectivos para analisar as características clínicas e o curso temporal da disfunção neurológica após a infecção grave por SARS-CoV-2. Várias revisões sistemáticas com metanálises estão disponíveis sobre esse tópico;^(7-9)^ no entanto, ainda são necessários estudos controlados e randomizados para definir as melhores estratégias de reduzir o impacto da doença.^(7)^

Os autores também acreditam que a pesquisa minuciosa de disfunção neurológica em pacientes críticos (inclusive excedendo a população de SARS-CoV-2) é de fundamental importância. De fato, todos os pacientes incluídos no estudo foram submetidos a exames neurológicos diários durante sua internação na UTI, conforme o padrão de prática clínica do Departamento de Medicina Intensiva de nosso centro, em conformidade com diretrizes internacionais.^(10)^

Com relação aos pontos observados pelos Drs. Finsterer e Scorza^(11)^ e Magoon e Suresh,^(12)^ esses tópicos foram abordados em suas respectivas cartas-resposta.^(13,14)^

Reconhecemos as limitações de nosso estudo, conforme relatado na seção Discussão de nosso artigo, razão pela qual a principal mensagem a ser considerada nele é a necessidade de rastreio sistemático de complicações neurológicas em pacientes com SDRA, particularmente naqueles com insuficiência respiratória aguda associada à COVID-19.^(2)^

Os autores agradecem ao Dr. Villa^(1)^ por esta discussão relacionada ao nosso artigo num tópico específico em que todos concordamos: a importância de avaliar e tratar a disfunção neurológica relacionada à COVID-19.
